# Targeting Hepatic Stellate Cells for the Prevention and Treatment of Liver Cirrhosis and Hepatocellular Carcinoma: Strategies and Clinical Translation

**DOI:** 10.3390/ph18040507

**Published:** 2025-03-31

**Authors:** Hao Xiong, Jinsheng Guo

**Affiliations:** 1 Department of Gastroenterology and Hepatology, Zhongshan Hospital, Shanghai Institute of Liver Diseases, Fudan University, Shanghai 200032, China; xiongh039@163.com; 2 Department of Internal Medicine, Shanghai Medical College, Fu Dan University, Shanghai 200032, China

**Keywords:** hepatic stellate cells, liver cirrhosis, hepatocellular carcinoma, targeting therapeutics

## Abstract

Hepatic stellate cells (HSC) are the major source of myofibroblasts (MFB) in fibrosis and cancer- associated fibroblasts (CAF) in both primary and metastatic liver cancer. Over the past few decades, there has been significant progress in understanding the cellular and molecular mechanisms by which liver fibrosis and HCC occur, as well as the key roles of HSC in their pathogenesis. HSC-targeted approaches using specific surface markers and receptors may enable the selective delivery of drugs, oligonucleotides, and therapeutic peptides that exert optimized anti-fibrotic and anti-HCC effects. Recent advances in omics, particularly single-cell sequencing and spatial transcriptomics, hold promise for identifying new HSC targets for diagnosing and treating liver fibrosis/cirrhosis and liver cancer.

## 1. Introduction

**Figure 1:**
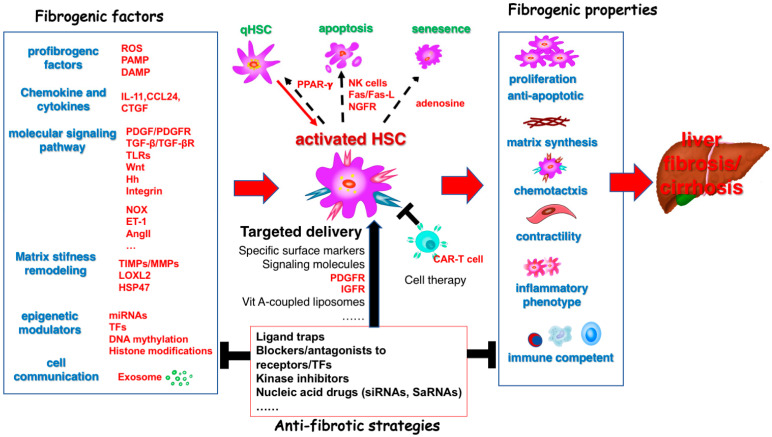
Targeting hepatic stellate cell activation for the treatment of liver fibrosis and cirrhosis. Activated hepatic stellate cells (HSC) play a central role in the development and progression of liver fibrosis and cirrhosis. Various fibrogenic factors trigger the transition of quiescent hepatic stellate cells (qHSC) into activated HSC, which acquire multiple fibrogenic properties. Anti-fibrotic strategies are designed to target HSC and block their activation by fibrogenic factors, as well as their fibrogenic properties once activated. Red arrows (→): Indicate the promoting effect and fibrosis/cirrhosis progression. Black T-bar (⊥): Represents inhibitory or blocking effects. Abbreviations: AngII, Angiotensin-II; CAR-T cell, Chimeric antigen receptor T cell; CCL24, C-C motif chemokine ligand 24; CTGF, Connective Tissue growth factor; DAMP, Damage-associated molecular patterns; ET-1, Endothelin-1; Fas/Fas-L: Fas cell surface death receptor/Fas ligand; Hh: Hedgehog; HSC, Hepatic stellate cell; HSP47: Heat shock protein 47; IGFR, Insulin-like growth factor receptor; IL-11, Interleukin 11; LOXL2, Lysyl oxidase-like 2; MMPs, Matrix metalloproteinases; miRNAs: microRNAs; NGFR: Nerve growth factor; NK cells: natural killer cells; NOX, NADPH oxidase; PAMP, Pathogen-associated molecular patterns; PDGF, Platelet-derived growth factor; PDGFR, Platelet-derived growth factor receptor; PPAR-γ, Peroxisome proliferator-activated receptor gamma; qHSC, Quiescent hepatic stellate cell; ROS, Reactive oxygen species; SaRNAs, Small activating RNAs; siRNAs, Small interfering RNAs; TFs: Transcription factors; TGF-β, Transforming growth factor beta; TGF-βR, Transforming growth factor beta receptor; TIMPs, Tissue inhibitors of metalloproteinases; TLRs, Toll-like receptors; Wnt: Wingless and Integration.

**Figure 2:**
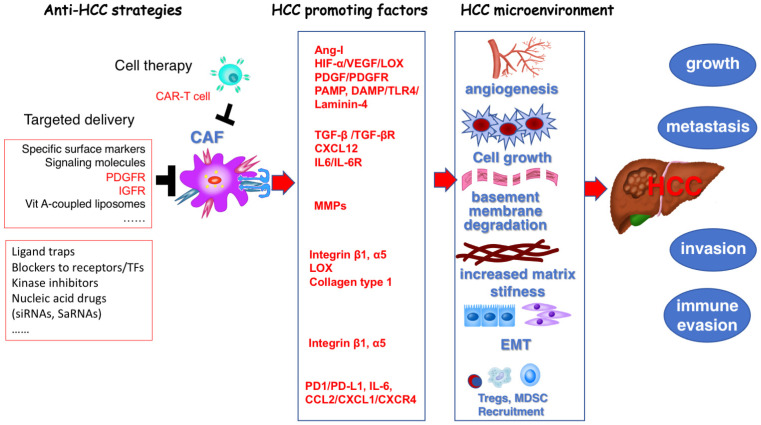
Targeting cancer-associated fibroblasts for the treatment of hepatocellular carcinoma. Cancer-associated fibroblasts (CAF) secrete various hepatocellular carcinoma (HCC)-promoting factors that shape the HCC microenvironment to foster tumor growth, metastasis, invasion, and immune evasion. Red arrows (→): Indicate the promoting effect of HCC development and progression. Black T-bar (⊥): Represents inhibitory or blocking effects. Abbreviations: Ang-I, Angiotensin I; CAF, Cancer-associated fibroblast; CAR-T cell, Chimeric antigen receptor T cell; CCL, C-C Motif Chemokine Ligand; CXCL, C-X-C Motif chemokine ligand; CXCR4, C-X-C Motif chemokine Receptor 4; DAMP, Damage-Associated Molecular Patterns; EMT, Epithelial–Mesenchymal Transition; HCC, Hepatocellular Carcinoma; HIF-α, Hypoxia-inducible factor alpha; IL-6, Interleukin 6; LOX, Lysyl oxidase; MDSC, Myeloid-derived suppressor cells; MMPs, Matrix metalloproteinases; PAMP, Pathogen-associated molecular patterns; PD1, Programmed cell death protein 1; PD-L1, Programmed death ligand 1; TGF-β, Transforming growth factor beta; TGF-βR, Transforming growth factor beta receptor; Tregs, Regulatory T cells; VEGF, Vascular endothelial growth factor.

Approximately 844 million people worldwide suffer from chronic liver diseases (CLD), resulting in approximately 2 million deaths annually, a trend that continues to rise [1,2]. Liver fibrosis is a scar tissue repair response following chronic liver injury from various etiologies. The persistent accumulation of scar tissue leads to irreversible changes in liver structure and function, resulting in decompensated cirrhosis, complications of portal hypertension, and a significantly increased risk of hepatocellular carcinoma (HCC). There is an urgent need for effective prevention and early diagnosis to manage and treat CLD, thereby preventing cirrhosis-related morbidity and mortality. Current treatments for liver fibrosis primarily focus on addressing the underlying causes of liver disease, such as inhibiting hepatitis B virus (HBV) and hepatitis C virus (HCV) replication or suppressing immune responses in autoimmune hepatitis. Direct anti-fibrosis treatments are still lacking due to the low bioavailability of most therapeutic agents and their insufficient capacity for hepatic targeting and accumulation, as well as frequent adverse reactions. The anti-fibrosis field faces significant challenges in mechanistic understanding, therapeutic targeting, and clinical translation. Hepatic stellate cells (HSC) are the major source of myofibroblasts (MFB) in fibrosis and cancer-associated fibroblasts (CAF) in both primary and metastatic liver cancer [3,4]. Targeting the activation of HSC is important not only for fibrosis treatment (Figure 1) but also for altering their interaction with HCC cells, which is also a strategy for HCC treatment (Figure 2).

## 2. The Role of Hepatic Stellate Cells (HSC) in Liver Cirrhosis and HCC

### 2.1. HSC and Liver Fibrosis

HSC are non-parenchymal cells in the liver that play an essential role in fibrogenesis and the progression of liver cirrhosis. The activation of HSC, transitioning from quiescent, fat-storing (retinyl ester) pericytes residing in the liver’s peri-sinusoidal space to persistently activated myofibroblasts, is a critical event in the development of liver fibrosis [5]. Under stimuli such as cell death (apoptosis and necrosis), inflammation, oxidative stress, extracellular matrix degradation and remodeling, and pro-fibrotic cytokines, HSC are activated and transdifferentiated into MFB phenotypes characterized by high proliferative activity. They lose their characteristic lipid droplets containing retinoids, express α-smooth muscle actin (α-SMA) and extracellular matrix (ECM) components such as collagen, and acquire contractility, chemotaxis, and migratory capabilities. Activated HSC also produce large amounts of growth factors and pro-fibrotic cytokines, including transforming growth factor β (TGF-β) and platelet-derived growth factor (PDGF). Furthermore, activated HSC exhibit an inflammatory phenotype, releasing pro-inflammatory cytokines such as monocyte chemoattractant protein-1 (MCP-1), which promotes the accumulation of inflammatory cells and amplifies the inflammatory response [6].

### 2.2. CAF and HCC

As a predominant complication of cirrhosis, liver cancer is characterized by crucial alterations in inflammation and fibrosis/cirrhosis within the microenvironment, with HSC as the main source of CAF in the HCC stroma [7]. CAF can be located in the sinusoids, fibrous septa, and capsule of liver tumors, as well as within the tumor matrix. These cells increase matrix stiffness, enhance ECM remodeling, and secrete growth factors, chemokines, angiogenic factors, tumor-promoting microRNAs, and exosomes that are taken up by tumor cells, supporting and facilitating HCC tumorigenesis, survival, growth, and metastasis [8,9,10]. A combined single-cell RNA sequencing (scRNA-seq) analysis with genetic tools to activate, deplete, inhibit, and manipulate HSC and their mediators during HCC development revealed that there is a progressive imbalance of HSC subpopulations during disease progression. Quiescent HSC, which express protective factors, shift toward activated HSC, which express tumor-promoting mediators and secrete factors (e.g., VEGF and angiopoietin-1) that promote angiogenesis, thereby setting the stage for HCC development in CLD.

CAF may also play a key role in inducing immune tolerance and immune evasion in HCC cells [11,12]. HCC CAF modulates antigen presentation by dendritic cells (DC), recruits myeloid-derived suppressor cells (MDSC) and tumor-associated neutrophils, and recruits tumor-associated macrophages while inducing their polarization. HCC patients with a high density of CAF around the tumor have a poor long-term clinical prognosis and a high incidence of early recurrence and metastasis. The density of Foxp3^+^ regulatory T cells (Treg) and CD68^+^ macrophages around the tumor is positively correlated with the density of CAF. Activated HSC accelerate activated T cell apoptosis, increase Treg numbers, and inhibit T cell-mediated cytotoxicity. Activated HSC inhibit T cell function by upregulating programmed death ligand PD-L1 (B7-H1) to bind to its receptor PD-1. PD-L1 expressed by HSC can induce T cell apoptosis, attenuate T cell infiltration, and suppress T cell-mediated cytotoxicity. HSC activation causes massive production of Th2/Th3-like cytokines, downregulation of Th1-like cytokines and cytotoxic T cell (CTL) function, and further suppression of the host immune system. aHSC also induce the expansion of two suppressive immune cell populations: myeloid-derived suppressor cells (MDSCs) and T helper 17 (Th17) cells, a subset of CD4^+^ effector T cells. Some cytokines secreted by HSC, such as IL-6, may also promote MDSC and inhibit T cell proliferation and function.

## 3. Targeting HSC for the Prevention and Treatment of Liver Cirrhosis

The activation of HSC is a determining factor in liver fibrosis progression and a critical target cell for anti-fibrotic treatments in various liver diseases [13,14]. Inhibiting stellate cell proliferation, differentiation, and activation is an attractive strategy for ameliorating hepatic fibrosis. This may be achieved by blocking the synthesis of key molecules involved in fibrogenesis with nucleic acid drugs (e.g., siRNA, saRNA), blocking ATP binding to the receptors and transcription factors of fibrogenic signaling pathways with kinase inhibitors, and preventing key ligands from binding to their receptors using monoclonal antibodies and high-affinity ligand traps (Figure 1). Multiple signaling pathways and downstream transcription factors mediate the pro-fibrotic effects of HSC in liver fibrosis, including PDGF, integrin, TGF-β, and Toll-like receptor 4 (TLR4) signaling pathways [15,16,17,18]. Various signaling molecules also contribute to fibrogenesis, such as platelet-derived growth factor (PDGF), TGF-β, connective tissue growth factor (CTGF, or CCN2), damage-associated molecular patterns (DAMP), and pathogen-associated molecular patterns (PAMP) [19,20]. Additional mediators that promote HSC activation include angiotensin, leptin, osteopontin, and hedgehog ligands [21,22]. The representative clinical trials of potential anti-fibrotic therapies in chronic liver diseases are shown in Table 1. Among the promising targets and pathways, such as TGF-β, PDGF, and integrin, that have been tested in clinical trials, the closest prospects include an integrin antagonist (PLN-74809) in clinical trials for PSC (ClinicalTrials.gov ID NCT04480840) and Hydronidone, which inhibits TGF-β signaling and modulates pro-inflammatory pathways (NCT05115942). They are undergoing further trials to assess long-term outcomes and the durability of anti-fibrotic effects. Furthermore, given that liver fibrosis arises from diverse etiologies (e.g., viral hepatitis, metabolic dysfunction, alcohol-induced injury), targeting the specific etiology of liver diseases to inhibit the generation of fibrogenic factors should also be considered important for combination therapies. This includes targeting the glucagon-like peptide-1 (GLP-1) receptor (Semaglutide, NCT04822181) and the thyroid hormone receptor-beta (THRβ) (Resmetirom, NCT03900429) in the context of metabolic dysfunction-associated steatohepatitis (MASH).

### 3.1. Molecular Signaling Pathways Associated with HSC/MFB Activation

#### 3.1.1. Reactive Oxygen Radicals (ROS)

In the liver, ROS primarily originate from injured hepatocytes. Additionally, non-parenchymal cells, such as activated HSC and Kupffer cells, contribute to ROS production. Excessive production of intrahepatic ROS during liver injuries activates HSC and promotes the progression of liver fibrosis [23]. ROS activate signaling pathways and transcription factors, such as JNK and NF-κB, and increase the expression of MCP-1, Col-1, and TIMP-1. ROS is produced by mitochondrial damage and the activation of cytochrome P450 (especially cytochrome P450 2E1), xanthine oxidase, and NADPH oxidase (NOX). NOX is a group of enzymes with seven known homologs, and the activation of NOX1, NOX2, and NOX4 plays a key role in the activation of HSC and is considered a therapeutic target [24,25].

#### 3.1.2. Toll-like Receptors (TLRs)

TLR4 is expressed by activated HSC and hepatic immune cells and is often a cofactor in the development and progression of liver fibrosis. TLR4 can be activated by exogenous PAMP ligands, such as lipopolysaccharides from intestinal bacteria, and endogenous DAMP, such as high mobility group box-1 (HMGB1). The activation of TLR4 enhances TGF-β1 signaling and promotes the fibrogenic effects of inflammatory chemokines (such as CCL2 and IL-6) by downregulating the expression of osteoblastin and the TGF-β1 transmembrane inhibitor (BAMBI) [26]. Therefore, TLR4 inhibition has been considered in clinical trials for the treatment of liver fibrosis in MASH (ClinicalTrials.gov IDs: NCT02442687, NCT04255069). In addition, TLR3 promotes liver fibrosis activity by stimulating IL-10 production.

#### 3.1.3. Hedgehog Signaling Pathway

The hedgehog (Hh) pathway is a highly conserved signaling pathway that has been implicated in cell proliferation, adhesion, migration, differentiation, and embryogenesis. In the liver, Hh signaling in HSC is pro-fibrotic, mediated through multiple downstream transcriptional targets, including Smo, Ptc, Gli1, and Gli2. Hh signaling promotes the activation of HSC and liver repair [27]. The interaction of the Hh and Notch signaling pathways regulates key cells in the liver repair process by regulating epithelial–mesenchymal transition (EMT). The Hh pathway also regulates metabolic pathways (e.g., glycolysis and glutaminolysis) [28], as well as DNA methylation and DNA methyl-binding protein (MeCP2). Forskolin, an Hh signaling inhibitor, can alleviate the progression of CCl4-induced murine liver fibrosis [29]. Ligustrazine plays an anti-fibrotic role by blocking Hh signaling, which arrests the cell cycle and triggers apoptosis of HSC. Curcumin induces apoptosis of HSC and regulates glycolysis and other metabolic pathways of HSC by inhibiting the Hh signaling pathway [30]. Therefore, specific Hh signal inhibitors can be used as promising therapeutic agents for anti-liver fibrosis treatment.

#### 3.1.4. Wnt Signaling Pathway

The Wnt signaling pathway can be classified into classical (i.e., Wnt/β-catenin) and non-canonical Wnt signaling pathways, which involve proteins such as Wnt5a and Wnt11. The Wnt signaling pathway is involved in cell proliferation, differentiation, and apoptosis, promoting HSC activation and inhibiting apoptosis [31]. The classical Wnt signaling pathway inhibits the activation of HSC by increasing MeCP2 protein expression, which subsequently inhibits peroxisome proliferator-activated receptor γ (PPAR-γ) [32]. MiR-17-5p has been implicated in liver fibrosis progression by inhibiting Wnt inhibitory factor 1 (WIF1) expression and activating the Wnt/β-catenin signaling pathway [33]. Hesperetin derivative-7 (HDND-7) reverses liver fibrosis by modulating the Wnt/β-catenin signaling pathway, thereby inhibiting HSC activation and proliferation [34]. Wnt5a may be involved in the regulation of inflammatory cytokine production in HSC and cell proliferation, making it a potential therapeutic target for liver fibrosis [35].

#### 3.1.5. Fibroblast Growth Factor (FGF)/Fibroblast Growth Factor Receptor (FGFR) Signaling Way

FGF1 and FGF2 serve as important regulators of HSC activation with regard to collagen synthesis. FGF1 inhibits the TGF-β-induced activation of HSC [36], and FGF2 promotes the inactivation of HSC to a more quiescent-like phenotype [37]. However, the combined loss of both FGF1 and FGF2 also provides protection against hepatic fibrosis, suggesting that the effects of endogenous FGFs and exogenous FGFs may differ [38,39]. FGF21 suppresses HSC activation by inhibiting the TGF-β and NF-κB signaling pathways [40]. PsTag-FGF21, a kind of long-acting FGF21 analog, modulates the macrophage Ly6C phenotypic switch by upregulating NR4A1. NR4A1 transcriptionally regulates the secretion of IGF-1 from Ly6Clo macrophages, thereby inhibiting HSC activation [41].

#### 3.1.6. Thyroid Hormone Receptor (THR)

THRs, including THR-α and THR-β, are expressed in the liver and are responsible for the regulation of glucose and fatty acid metabolism [42]. In HSC, THR-α is the predominant subtype. Both of them down-regulate during hepatic fibrogenesis in humans [43]. Studies suggest that THRs contribute to the modulation of HSC activation. In a MCD model, THR-α-deficient mice demonstrate exacerbated liver fibrosis, while triiodothyronine (T3) administration suppresses TGF-β-mediated HSC activation and lowers phosphorylated Smad2 levels.

Resmetirom, which selectively activates THR-β, is the first drug recently approved for the treatment of metabolism-associated fatty liver disease (MAFLD). It has demonstrated efficacy in reducing hepatic fat accumulation, improving liver histology, and alleviating biomarkers of liver damage without significantly disturbing glucose metabolism or body weight [44].

#### 3.1.7. Chemokine and Cytokine

In addition to TGF-β, a number of other growth factors and chemokine targets are being pursued, including interleukin (IL)-11, connective tissue growth factor (CCN2), and CCL24. Interleukin 11 (IL-11) exhibits remarkably pleiotropic activity on epithelial cells and mesenchymal cells across a number of tissues, including the liver. Antagonism or knockout of IL-11 attenuates HSC activation while reducing steatosis and metabolic derangements within hepatocytes in MASH [45]. A neutralizing antibody to IL-11 has been evaluated in a clinical trial (NCT05658107) for its efficacy and safety in MASH. CCL24 is a circulating chemokine produced by epithelial cells and fibroblasts that binds to its cognate receptor CCR3 to promote inflammation, cell trafficking, and fibrosis [46]. Serum levels of CCL24 correlate with the severity of fibrosis and are particularly elevated in patients with primary sclerosing cholangitis. CCL24 levels also correlate with disease stage in systemic sclerosis [47]. A monoclonal antibody targeting CCL24 has demonstrated efficacy in several animal models of liver disease, leading to its evaluation in early clinical trials [48]. A completed Phase 1B trial in patients with MASLD but not MASH demonstrated good tolerability and improvement in several serum markers of collagen turnover and inflammation, and a Phase 2A randomized, placebo-controlled trial is currently underway (NCT05824156).

### 3.2. Molecular Signaling Pathways Associated with HSC/MFB Proliferation

PDGF is the most potent proliferative stimulus for HSC. PDGF belongs to a family of growth factors consisting of four secreted extracellular ligands encoded by four different genes. They assemble into disulfide-bonded dimers via homo- or heterodimerization. All members share a highly conserved and specific PDGF/VEGF homology domain necessary for receptor binding and activation. The PDGF ligands exert their biological effects through two structurally related tyrosine kinase receptors, PDGFR-α and PDGF-β. PDGF-β is upregulated during HSC activation both in vitro and in vivo. PDGF-B and -D bind to PDGF-β, resulting in downstream phosphorylation of extracellular signal-regulated protein kinase/mitogen-activated protein kinase (Erk/MAPK) and protein kinase B (Akt/PKB) in the phosphoinositide-3-kinase (PI3K) pathways, leading to significant HSC proliferation.

Blocking the biological effects of PDGF inhibits HSC proliferation and reduces the degree of liver fibrosis. Imatinib mesylate (Gleevec), a clinically used PDGF receptor (PDGFR) tyrosine kinase inhibitor, could be a promising molecular targeted approach to limit the liver fibrosis development. PDGF antagonists, PDGF-specific neutralizing antibodies (e.g., AbyD3263, MOR8457), and soluble dominant-negative PDGF receptor inhibitors can serve as targeted therapies to block PDGF. In addition, several multi-kinase inhibitors targeting PDGF receptors (e.g., imatinib, nilotinib, sorafenib) are currently undergoing clinical trials [49]. Although extensive efforts have been made to study PDGF systems in liver fibrosis, most PDGF antagonists that block fibrogenic activation and ECM production of MFB work well in culture and in some rodent models of liver fibrosis but carry a high risk of unwanted side effects in patients due to their lack of specificity. Inhibition of PDGFR-β signaling with tyrosine kinase inhibitors has proven effective in early phases but not in advanced cirrhotic models in rodents. Nevertheless, they remain among the more promising developments due to their longstanding use in cancer and established safety and tolerability.

### 3.3. Molecular Signaling Pathways Associated with Pro-Liver Fibrosis

Anti-fibrotic targets may be conserved across multiple organs. The concept of core and regulatory pathways has been proposed to identify optimal targets for anti-fibrotic drug discovery/design. Core pathways are conserved in different organs and species, whereas regulatory pathways may be confined to specific cell types or organs. Potential core pathway components include lysyl oxidase 2 (LOXL2), αv-integrin, TGF-β, and their downstream signaling effector molecule CTGF, and drugs targeting these molecules are currently being evaluated in clinical trials.

#### 3.3.1. Transforming Growth Factor β (TGF-β)

The TGF-β pathway plays a crucial role in maintaining physiological homeostasis, including immunomodulation and tumor suppression. It has long been recognized as the most potent driver of fibrosis in all tissues and remains the primary anti-fibrotic target in HSC and other fibrogenic cell populations [50]. Blocking the TGF-β1 pathway inhibits liver fibrosis development; however, the systemic inhibition of TGF-β1 can promote inflammation and adversely affect liver parenchyma and precursor cells. Integrins αvβ6 and αvβ8 play a key role in activating latent TGF-β1 [51,52]. Therefore, small molecule integrin inhibitors and function-blocking antibodies may serve as therapeutic strategies for liver fibrosis treatment.

However, the pleiotropic effects, multiple activation modes, and diverse signaling pathways of TGF-β, which are cell type- and cell state-specific, have made this a challenging target. Moreover, systemic antagonism of TGF-β is unsafe, as inhibiting its developmental, antiproliferative, antiapoptotic, and anti-inflammatory activities can disrupt tissue homeostasis and promote cancer [53,54,55]. Therefore, TGF-β antagonists are being sought that antagonize only its fibrogenic activity while preserving other functions. One strategy seeks to inhibit cell surface integrins that contribute to TGF-β activation at the cell membrane. This approach underlies the promise of using a small molecule (Bexotegrast, PLN-74809) that blocks the activity of αvβ1 and αvβ6 in pulmonary fibrosis [56] and primary sclerosing cholangitis (NCT04480840). An exciting new approach has leveraged the discovery that latent TGF-β is complexed with different proteins, each of which mediates different activities of the cytokine. Specifically, whereas release of latent TGF-β from either GARP or LRRC33 largely regulates its immunogenic activity [57,58], its binding to latent TGF-β binding protein (LTBP) controls its fibrogenic activity [59]. With this knowledge, investigators have developed an antibody that only prevents the release of LTBP-bound TGF-β but does not block the release of TGF-β from GARP or LRRC33, thereby inhibiting fibrosis while preserving TGF-β’s immunoregulatory and other activities [60]. As proof of principle, this antibody attenuates the progression of renal fibrosis in two mechanistically distinct mouse models; however, no studies in MASH models have been reported yet. Another approach to antagonizing TGF-β activity distinguishes between the differential fibrogenic activities of the three major TGF-β isoforms, TGF-β1, TGF-β2, and TGF-β3. A recent study indicates that most of TGF-β’s fibrogenic activity can be attributed to TGF-β2 and TGF-β3 [61], whose antagonism avoids the liabilities of inhibiting TGF-β1; this strategy shows promise in systemic sclerosis but has not yet been explored in liver fibrosis [62].

Connective tissue growth factor (CTGF) is a molecular downstream of TGF-β1 signaling that amplifies its signaling effect, and a monoclonal antibody to CTGF (FG-3019) completed clinical trials II but failed to show efficacy in Phase 3 trials against pulmonary fibrosis (NCT01890265, NCT04419558) [63,64].

#### 3.3.2. FAP

Activated hepatic stellate cells adopt a more myofibroblast-like phenotype and express α-smooth muscle actin (αSMA), glial fibrillary acidic protein (GFAP), and fibroblast activation protein (FAP) [65]. Intrahepatic expression of FAP, but not GFAP or αSMA, correlated with the degree of liver fibrosis in patients with hepatitis C virus (HCV) infection [66]. FAP activity was 14- to 18-fold higher in cirrhotic livers compared to healthy livers, and circulating FAP levels nearly doubled in patients with alcoholic cirrhosis. The concentration and activity of circulating FAP were significantly increased in patients with liver cirrhosis compared to those with healthy livers, and these elevated levels correlated with increased cleavage of α-2 antiplasmin.

Inhibition of FAP with a highly selective inhibitor can nearly restore the inflammatory response and markedly decrease the activation of HSC, as well as the synthesis and accumulation of collagen in advancing parenchymal liver fibrosis [67]. This effect is also associated with a reduction in the total number of macrophages, particularly M2-type macrophages. N-terminally cleaved α-2 antiplasmin is a more potent inhibitor of fibrinolysis than its uncleaved form. Therefore, it has been proposed that elevated circulating FAP may contribute to hemostasis-related bleeding and thrombotic events in liver cirrhosis. Low levels of circulating FAP can be used clinically to rule out significant liver fibrosis in patients with nonalcoholic fatty liver disease [68].

#### 3.3.3. Cannabinoid Receptors (CB)

The cannabinoid receptors CB1 and CB2 are components of the G-protein-coupled receptor family and the endocannabinoid system, both of which play key roles in the progression of liver fibrosis in chronic liver disease. The active component of *Cannabis sativa* inhibits the growth and promotes the apoptosis of hepatic stellate cells through CB2 receptors. Activation of CB2 receptors by anandamide also prevents the excessive proliferation of cholangiocytes, which is a common consequence of extrahepatic biliary obstruction, cholestasis, and toxic liver damage [69]. Activated HSC express CB1 receptors, which promote fibrosis. The first-generation CB1 antagonist rimonabant was withdrawn from clinical use due to its association with depression. Currently, peripherally restricted CB1 antagonists that do not cross the blood–brain barrier are under development. CB2 receptors are currently regarded as promising targets for anti-inflammatory and anti-fibrotic therapy, and treatment with CB1 antagonists and CB2 analogues may represent an ideal multi-target anti-fibrotic approach [70,71].

### 3.4. Molecular Signaling Pathways Associated with HSC/MFB Contraction Responses

Endothelin-1 (ET-1) is a potent hepatic vasoconstrictor [72]. In a healthy liver, ET-1 is primarily produced by endothelial cells, whereas in liver injury, it is predominantly produced by hepatic stellate cells (HSC). Activated HSC exhibit high expression of ET-1, angiotensin-II (Ang-II), and their receptors. The renin-angiotensin system (RAS) regulates the formation of liver fibrosis. Blocking RAS through angiotensin receptor 1 (AT1) blockers and angiotensin-converting enzyme inhibitors (ACE inhibitors) may be an effective strategy for treating liver fibrosis. There is crosstalk between the Ang-II/AT1 and ET-1 systems, with Ang-II inducing the expression of ET-1 in HSC via the PI3K/Akt signaling pathway, and ET-1 promoting the role of Ang-II in the transdifferentiation of HSC into MFB-like cells [73].

### 3.5. Molecular Signaling Pathways Associated with Reversal of Liver Fibrosis

#### 3.5.1. Activated HSC Return to Resting Molecular Signaling Pathways

PPAR-γ is a nuclear transcription factor that can be activated by peroxisome proliferators and plays an important role in maintaining HSC in a quiescent state. Reduced PPAR-γ expression promotes HSC activation, while PPAR-γ agonists or ligands can inhibit HSC activation and reduce ECM deposition, making them potential targets for anti-fibrotic therapy [74]. The combination of the PPAR-α/δ stimulant (GFT505) has been shown to have significant hepatoprotective effects in animal models of nonalcoholic fatty liver disease, making it a highly promising direction for MAFLD/MASH-targeted drug therapy [75].

#### 3.5.2. Molecular Signaling Pathways That Induce the Apoptosis and Senescence of Activated HSC/MFB

Inducing apoptosis in activated HSC is an important strategy for liver fibrosis regression, and HSC contain multiple molecular families that mediate apoptosis, such as Fas/Fas-L, NF-κB, nerve growth factor receptor, and Bcl-2/Bax. These pathways can be studied in depth and applied to the treatment of anti-liver fibrosis. NK cells are generally considered to have anti-fibrotic therapeutic potential because they promote activated HSC apoptosis through TRAIL/DR5 and NKG2D-RAE1 [76].

Senescent HSC are a proposed target for anti-fibrotic therapies, based on their reported roles in hepatic fibrosis, MASH, and HCC development. The core features of senescence are irreversible growth arrest and increased expression of β-galactosidase (SA-Bgal), which are associated with cell morphology and senescence. The senescence mechanisms of HSC are mainly governed by the p16-Rb and Arf-p53-p21 pathways. The adenosine A2A receptor can promote the progression of liver fibrosis by reducing p53 and Rb through the PKA/Rac1/p38 MAPK pathway, thereby reducing HSC senescence and promoting HSC proliferation, which provides strong evidence for adenosine’s role in regulating the progression of liver fibrosis [77].

Integrated high-resolution single-nucleus RNA sequencing, combined with immunostaining and flow cytometry, has identified the urokinase plasminogen activator receptor (uPAR) as a marker of senescent HSC. uPAR is expressed by activated HSC during early injury and by immune cells as liver injury progresses [78]. Senescent HSC can be selectively eliminated using senolytic CAR T cells that target uPAR or other senescence-associated proteins, such as the mannose receptor CD206, to reduce liver fibrosis. HSC-derived CAF might be depleted by targeting FAP via DNA vaccines, CAR T cell therapy, or oncolytic viruses, potentially reducing hepatic fibrosis and tumor burden.

Activated HSC exhibit elevated expression of αV integrin and TGF-β receptor, both of which are potential therapeutic targets for fibrosis reduction. A small molecule, CWHM12, pharmacologically blocks αV integrin to attenuate fibrosis [79], and TGF-β receptor signaling can also be locally inhibited by targeting caveolin, hyaluronic acid synthase 2, CD147, hydrogen peroxide-inducible clone 5, or galectin-1. After prolonged activation, HSC can senesce and secrete the SASP component IL-33 via the gasdermin D pore, which promotes tumor development [80]. Disulfiram reduces tumor burden in mice by inhibiting the gasdermin D pore [81].

### 3.6. ECM and Liver Fibrosis

Liver fibrosis is a dynamic process resulting from an imbalance between ECM synthesis and degradation. The regulation of ECM is primarily controlled by the balance between matrix metalloproteinases (MMPs) and matrix metalloproteinase inhibitors (TIMPs). Enhancing MMP activity while reducing TIMP activity promotes ECM degradation and suppresses ECM synthesis, contributing to anti-fibrotic effects [82]. HSC are the main source of MMP-2, MMP-9, and MMP13. MMP-2 inhibits collagen I production and promotes HSC apoptosis via cadherin signaling. HSC are also the main source of TIMPs, and TIMP1 is an anti-apoptotic factor in activated HSC; therefore, TIMP1 can be used as one of the key targets for the treatment of liver fibrosis.

In addition, osteopontin, an ECM cytokine expressed by HSC, promotes collagen I expression through integrin αvβ3 and the activation of the PI3K/pAkT/NFκB signaling pathway [83], thereby promoting ECM synthesis and increasing hepatic fibrosis.

Lysyl oxidase-like 2 (LOXL2) plays a role in matrix stiffness and collagen cross-linking [84]. Moreover, LOXL2 has been shown to inhibit ECM degradation in animal models of CCl4-induced liver fibrosis, and humanized antibody inhibitors of LOXL2 have been tested in clinical trials for liver fibrosis [85,86]. Notably, phase 2 clinical trials targeting LOXL2 or the collagen chaperone heat shock protein 47 (HSP47) in HSC (NCT04267393) have not been successful in patients with end-stage MASH [87].

### 3.7. Epigenetic Regulation Associated with Liver Fibrosis

Genetic and epigenetic alterations in HSC create a self-reinforcing pro-fibrotic network. Targeting these mechanisms, particularly through HDAC inhibition, miRNA modulation, or metabolic reprogramming, holds significant potential to halt or reverse fibrosis.

#### 3.7.1. DNA Methylation and Related Histone Modifications

DNA methylation in HSC may contribute to the maintenance of their quiescent phenotype. Upon activation, HSC express MeCP2, which promotes the silencing of anti-fibrotic genes (e.g., IκB, PPARγ) and increases the expression of histone methyltransferases. This leads to enhanced transcription of COL-1, TIMP-1, and TGF-β. In mammals, five DNA methyltransferases (DNMTs) have been identified: DNMT1, DNMT2, DNMT3A, DNMT3B, and DNMT3L. In the CCl4-induced liver fibrosis model, DNMT1 was upregulated following HSC activation, while RNAi-mediated silencing of DNMT1 inhibited both HSC activation and proliferation [88]. Guadecitabine (SGI-110), a DNMT inhibitor and a novel hypomethylated dinucleotide of decitabine, is currently in Phase II clinical trials for hepatocellular carcinoma; however, it has not yet been tested for liver fibrosis [89]. In addition, histone deacetylase (HDAC) inhibitors have the potential to serve as therapeutic targets for liver fibrosis [90].

#### 3.7.2. MicroRNAs

MicroRNAs (miR) regulate HSC proliferation and fibrosis formation by modulating the expression of proliferative proteins and pro-fibrotic signaling pathways [91]. miR-27a, miR-27b, and miR-29b are significantly upregulated upon HSC activation. The physiological role of miR-29b is to inhibit the production of various extracellular matrix (ECM) proteins. Both TGF-β and lipopolysaccharide (LPS) regulate its expression levels, while hepatocyte growth factor (HGF) upregulates miR-29b expression. Additionally, the overexpression of miR-29b suppresses TGF-β-induced collagen production through the Smad signaling pathway [92]. The target molecules of miR-15b and miR-16 include Bcl-2. These miRNAs promote the expression of apoptosis-related proteins by downregulating Bcl-2 and accelerating the apoptosis of activated HSC. Recent studies have confirmed that miR-30 inhibits fibrosis by suppressing the expression of Kruppel-like factor 11 (KLF11) and attenuating TGF-β signaling in CCl4-induced animal models [93]. These findings provide new insights into the mechanisms that underlie liver fibrosis development.

### 3.8. Cell Therapies to Treat Fibrosis

CART cells are now being developed for a growing number of indications, including solid tumors, autoimmunity, and, most recently, senescence and fibrosis. To generate CART, DNA constructs encoding transmembrane chimeric receptors are transduced into T cells. Their general structure includes an antigen-binding domain on the cell surface linked to an intracellular domain that activates T cells upon ligand engagement [94]. Studies have implicated HSC as drivers of liver injury and inflammation, leading to efforts to identify cell surface markers of senescence in this cell type to target them for CAR T-mediated clearance. A pioneering study combined the knowledge of CART production with senescence biology to seek markers of senescence in HSC [95]. Using an informatics-based approach, the cell surface protein urokinase plasminogen activated receptor (uPAR) was identified as one such marker, and the administration of a uPAR-directed CAR T in a murine model of MASH attenuated fibrosis, cleared senescent cells, and improved serum albumin levels. Complementary to the CAR T approach for clearing senescent HSC, studies by Epstein and colleagues engineered CAR T to target only cells expressing FAP, which is a cell surface receptor that marks fibrogenic cells in several tissues, including the heart and joints, among others [96]. The administration of CAR T cells that were transduced ex vivo reduced the number of fibroblasts and fibrosis and improved cardiac function in a model of chronic cardiac injury [97]. These findings have established a target that does not rely on senescence and is more specific than uPAR. A remarkably elegant strategy by the same group built upon the conventional CAR T approach, instead developing a method for the in vivo programming of CAR T cells [98,99,100,101]. To do so, mRNA designed to program T cells into CAR T is delivered by lipid nanoparticles that target T cells in vivo, which instruct T cells to express CAR T directed to FAP on the fibroblast cell surface, yielding similar therapeutic benefits in the heart as conventional CAR T. This in vivo methodology has at least two distinct advantages. First, therapeutic CAR T-generating nanoparticles can be produced in advance and therefore be available immediately as an “off-the-shelf” technology, greatly expanding their availability beyond only facilities that can generate ex vivo CAR T onsite. Second, the use of RNA-expressing lipid nanoparticles avoids the integration of genetic material into the cell genome, thereby enabling titration of CAR T activity and allowing for discontinuation or repeat administration while avoiding unrestrained HSC clearance. Studies attempting to use in vivo CAR T targeting to FAP expressing cells in the liver are underway, complemented by studies using FAP imaging to quantify fibrosis [102]. While these reports underscore the potential benefit of selectively depleting HSC populations to reduce fibrosis, their complete elimination is potentially risky. A study in mice has demonstrated that when >99% of HSC are depleted using a cell therapy similar to CAR T, the livers fail to regenerate due to the loss of paracrine signals from HSC that support hepatocyte replication [103], highlighting the importance of HSC in maintaining liver homeostasis, as discussed above. Selective clearance of only those HSC populations that promote fibrosis may be a more rational approach than total HSC clearance.

## 4. Targeting Hepatic Stellate Cells for the Prevention and Treatment of Hepatocellular Carcinoma

### 4.1. Angiogenesis Provides Basic Survival and Metastatic Conditions for Tumor Cells

Angiogenesis is the main characteristic of HCC. Activated HSC promote the growth of the vascular epithelium by upregulating angiopoietin-I (Ang-I). aHSC secrete vascular endothelial growth factor (VEGF) to further promote angiogenesis. Additionally, the LPS/TLR4 pathway can induce HSC to secrete pro-angiogenic factors, which promote endothelial cell migration and tubulogenesis, thereby enhancing angiogenesis in HCC and providing nutrients for tumor cell growth [10]. The TLR4 signaling pathway in HSC promotes tumor progression and metastasis via the production of laminin-4 [9] and contributes to the formation of an immune-tolerant microenvironment in tumors.

In patients with cirrhosis, portal hypertension causes the translocation of gut microbiota to the liver. PAMP, such as lipopolysaccharides (LPS) derived from the outer surface membrane of Gram-negative bacteria, activate TLR4 in Kupffer cells and activated HSC. As a result, increased VEGF, Ang-I, and platelet-derived growth factor (PDGF) levels facilitate tumor angiogenesis. Additionally, the hypoxic environment resulting from liver inflammation, fibrosis, and rapid tumor growth promotes the expression of hypoxia-inducible factor 1 (HIF-1), thereby upregulating VEGF and promoting the formation of new vessels.

### 4.2. Matrix Stiffness Promotes Tumor Growth, Invasion, and Metastasis

Tumor cells exist in a favorable environment because of ECM deposits, collagen cross-linking, and increased matrix stiffness, which positively regulate tumor growth and metastasis. Cancer stem cells (CSC) originate from human stem cells and tumor cells. CSC, characterized by their ability to transform into different types of tumor cells, sustain the overall number of tumor cells through continuous proliferation and transformation. Increased matrix stiffness upregulates the stemness of HCC. Integrin β1 promotes the stemness of HCC cells by activating the serine-threonine protein kinase/animal rapamycin/sex-determining region Y-box 2 (AKT/mTOR/SOX2) pathway.

When comparing the effects of different mesenchymal stiffnesses on the epithelial–mesenchymal transition (EMT) and the invasive ability of tumor cells, higher matrix stiffness was found to promote tumor cell invasion and metastasis by upregulating EMT via integrins β1 and α5. Tumor progression may be related to the activation of TGF-β/Smad. Lysyl oxidase (LOX) increases matrix stiffness through collagen cross-linking. LOX is mainly involved in the EMT process and improves tumor invasion, which is reflected in patients with high LOX expression, who are more likely to relapse. HIF-1 can promote the expression of LOX. Researchers have found that LOX accelerates angiogenesis by upregulating angiogenic factors such as VEGF. Chemotherapy-resistant tumor cells can become tumor-initiating cells. These cells exhibit traits similar to CSC and often lead to tumor recurrence. These tumor-initiating cells secrete LOX, suggesting that LOX plays a crucial role in tumor progression and lesion formation.

### 4.3. Matrix Remodeling via MMP/TIMPs Are Crucial for Tumor Invasion and Metastasis

MMPs decompose components of the basement membrane and spread to other parts of the body, forming new lesions through blood circulation. Multiple studies have revealed that patients with HCC exhibit increased levels of MMP-2, MMP-4, MMP-9, and MMP-14. The expression level of MMP-9 correlates with HCC tumor cell invasion, potentially through MMP-mediated degradation of the basement membrane, which enhances the invasion of nearby blood vessels. Patients with high MMP-9 expression are more likely to experience relapse after radical resection.

### 4.4. Reprogramming of Cancer-Associated Fibroblasts

CAF, which are activated fibroblasts, regulate the biology of both stromal and tumor cells via cell–cell contact, release large amounts of cytokines and chemokines, and ultimately remodel the ECM. CAF secrete immunosuppressive ligands such as TGFβ and CXCL12. Reprogramming CAF with a TGFβ-R inhibitor, a CXCR4 blocker, or other methods increases T cell activation and infiltration while also decreasing CAF recruitment. This, in combination with immune checkpoint inhibitors (ICIs), may provide further clinical benefits and improve prognosis. It has shown better outcomes compared with monotherapy in basic and preclinical research, but more clinical trial data are needed to confirm these findings [104]. A combination of the PD-1 inhibitor Nivolumab and the TGF-β inhibitor Galunisertib (LY2157299) has been proposed in a clinical study (NCT02433343) involving HCC patients to determine the maximum tolerated dose (MTD) and progression-free survival (PFS).

## 5. Translational Barriers in Targeting HSC for Anti-Fibrosis and Anti-Tumor Therapy

### 5.1. Heterogeneity of HSC

The progression of liver fibrosis is a highly dynamic process with the potential for both progression and regression. HSC exhibit heterogeneity at different stages of liver fibrosis and cirrhosis progression and regression, as well as in the tumor microenvironment, where they function as CAF. This heterogeneity is reflected in varying activation phenotypes, differential expression of surface molecules, activation of signaling molecules, transcription factors, and the secretion of active molecules. Single-cell RNA sequencing (scRNA-Seq) studies have revealed that quiescent HSC form a highly homogeneous population, characterized by high expression of platelet-derived growth factor receptor (PDGFR). In contrast, in mouse livers with carbon tetrachloride-induced fibrosis, PDGFR-positive myofibroblasts can be divided into four distinct subpopulations, distinguished by the most significantly expressed marker genes [105]. Subpopulations MFB I, III, and IV express genes associated with collagen fiber formation, while MFB II expresses fewer matrix-related genes but more genes involved in the positive regulation of leukocyte activity and immune modulation. scRNA-Seq analysis also identified S100 calcium-binding protein A6 (S100A6) as a new marker specifically expressed in activated MFB but not in quiescent HSC. Furthermore, activated MFB secrete chemokines, such as CXCL1, which attract neutrophils from the circulation, whereas quiescent HSC and MFB express CCL2, a chemokine that recruits monocytes. The scRNA-Seq data confirm that the activation of HSC into MFB is a key feature of collagen expression, while quiescent HSC and selective MFB subpopulations secrete chemokines that regulate the inflammatory environment around them. With recent discoveries indicating that functionally and genetically distinct subtypes exist among activated HSC [106,107,108,109,110,111], efforts to target anti-fibrotic molecules to all activated stellate cells may not be as effective as selectively targeting only those subsets that clearly promote fibrosis. However, current strategies assume that most activated HSC share sufficient common features to make them viable therapeutic targets, a conclusion supported by a recent study documenting strong similarities in the activated HSC transcriptome across different etiologies of liver disease [112].

scRNA-Seq analyses have demonstrated the transition from quiescent HSC to several activated HSC and CAF phenotypes. In silico analyses describe the transcriptional differentiation of activated HSC into distinct subtypes, including proliferative (pHSC), inflammatory (iHSC), contractile/migratory (cmHSC), and fibrogenic myofibroblast (myHSC) phenotypes. Other subtypes expressing microvasculature genes have been identified within the vascular HSC (vHSC) subpopulation, yet their origins remain unclear. A deactivated HSC (dHSC) subpopulation emerges during liver fibrosis regression, characterized by an intermediate gene expression profile between quiescent and activated HSC. However, full reversion to the quiescent phenotype has not been observed. 

Similarly, CAF undergo a transcriptional transition from an inflammatory (iCAF) to a fibrogenic (myCAF) phenotype, with intermediary subpopulations, including vascular CAFs (vCAFs) and antigen-presenting CAFs (apCAFs). Type 1 collagen-expressing myHSC increase liver stiffness, thereby promoting tumor cell proliferation. Conversely, HSC expressing cytokines and growth factors (iHSC) suppress HCC growth via hepatocyte growth factor (HGF) and its receptor MET. In iCCA, myCAF produce hyaluronan synthase 2, the enzyme responsible for hyaluronic acid (HA) production, promoting tumor growth and progression. On the other hand, type 1 collagen produced by the myCAF subpopulation contributes to liver stiffness but does not affect tumor growth. The vCAF subpopulation promotes tumor growth via the interleukin (IL)-6/IL-6R axis. In liver metastasis, type 1 collagen derived from myCAFs suppresses tumor growth by mechanically restraining the tumor, whereas HA promotes tumor growth. In both iCCA and metastases, iCAF promote tumor growth via the HGF-MET axis. Due to the remarkable plasticity of HSC and CAF during liver fibrogenesis and carcinogenesis, their distinct subpopulations show complementary or ambiguous functions in response to specific chronic inflammatory and tumor microenvironments. Further identification of specific surface markers, signaling molecules, and therapeutic targets associated with liver fibrosis progression and regression, particularly in activated HSC, is needed. These could serve as both therapeutic targets and functional imaging biomarkers. Moreover, emerging evidence highlights that HSC heterogeneity and their potential adaptive mechanisms underpin resistance to current anti-fibrotic therapies, either through self-renewal, the compensatory upregulation of redundant signaling pathways, epigenetic plasticity, or metabolic reprogramming. Future studies may focus on the identification of resistance-associated subpopulations of HSC, and future anti-fibrotic strategies should emphasize combination therapies targeting multiple pathways (e.g., ECM remodeling, HSC activation, inflammation) and prioritize early-stage disease with robust biomarker-guided trial designs.

### 5.2. Lack of Specific Targeted Methods for HSC

Despite the identification of numerous anti-fibrotic candidate targets and the demonstration of potent anti-fibrotic effects in experimental animal models, their clinical efficacy remains limited or suboptimal. To date, no anti-fibrotic drugs have been approved for clinical use. The liver, characterized by its extensive vasculature and unique metabolic capacity, exhibits high overall drug uptake. However, most therapeutic agents are either metabolized by hepatic stem cells or cleared by the reticuloendothelial system, resulting in minimal drug delivery to HSC and consequently limiting therapeutic efficacy. Furthermore, the failure of many anti-fibrotic drugs in clinical trials is largely attributed to their low efficacy, which results from off-target uptake and rapid clearance. Consequently, the development of targeted drug delivery strategies that specifically activated HSC represents a critical approach to enhancing therapeutic responses across different stages of liver disease.

### 5.3. Barriers to the Translation of Basic Research into Clinical Practice

Significant progress has been made in elucidating the cellular and molecular mechanisms of liver fibrosis; however, only a few findings have been successfully translated into clinical applications. Firstly, the high cost of drug development and target validation necessitates prolonged timelines and substantial financial investment. Secondly, as regulatory requirements become more stringent, there is an increasing demand for drugs with well-defined clinical efficacy and safety profiles. Moreover, the efficacy observed in animal models often fails to fully translate to clinical settings due to differences in pharmacokinetics, extracellular matrix (ECM) cross-linking, and disease pathophysiology. Despite advancements in anti-fibrotic drug development, accurately identifying ideal noninvasive biomarkers for fibrotic activity and establishing consensus on optimal clinical endpoints remain significant challenges [113,114].

Currently, addressing the underlying cause remains the only proven strategy to halt or reverse liver fibrosis progression, while the development of effective anti-fibrotic therapies continues to pose a major challenge in liver disease management. Over the past few decades, substantial progress has been made in elucidating the cellular and molecular mechanisms underlying liver fibrosis. Liver fibrosis is a complex pathological change involving multiple cells, factors, and pathways, and the study of the cellular and molecular mechanisms of its occurrence and development provides an important theoretical basis and therapeutic target for clinical drug development. It is anticipated that improved animal models and well-designed clinical trials will facilitate the successful translation of anti-fibrotic research into effective clinical treatments in the near future.

## 6. Promoting Targeted Hepatic Stellate Cell-Based Strategies for the Prevention and Treatment of Liver Fibrosis and Hepatocellular Carcinoma

### 6.1. Utilizing New Omics Technologies to Identify Markers and Therapeutic Targets for Activated HSC

scRNA-Seq technology enables genome amplification of isolated single cells, followed by high-throughput sequencing after exonic capture, allowing for the identification of cell population heterogeneity and cellular evolutionary relationships. A key advantage of scRNA-Seq is its ability to generate multi-dimensional data, including differences in cell composition and abundance across tissues, gene expression variability, and functional alterations within seemingly homogeneous cell populations, as well as insights into cellular interactions and microenvironmental regulatory networks [115,116]. Integrating scRNA-Seq with spatial mapping has provided critical insights into the pathological and physiological mechanisms underlying liver fibrosis. This approach has been utilized to delineate metabolic zoning and molecular patterning in hepatocytes, endothelial cells, and HSC within human and murine liver lobules [117,118,119]. Single-cell analysis of human liver macrophage subpopulations has identified a novel scar-associated TREM2 + CD9+ macrophage subset, which originates from circulating monocytes and expands during liver fibrosis. These cells reside in the fibrotic microenvironment and promote mesenchymal cell activation and scar deposition [120]. Additionally, ACKR+ and PLVAP+ endothelial cells have been shown to expand during fibrosis, contributing to the establishment of the fibrotic microenvironment. Crosstalk among scar-associated macrophages, endothelial cells, and PDGFR+ collagen-producing mesenchymal cells reveals multiple fibrotic pathways, including the activation of TNFRSF12A, PDGFR, and NOTCH signaling. The interactions among these cells and subpopulations in liver fibrosis provide a molecular framework for targeting key pathogenic cell populations [121,122]. Moreover, fibrosis progression fingerprints derived from liver tissue and serum [123], along with dynamic network biomarkers (DNB) identified via systems biology approaches, offer promising targets for anti-fibrotic drug development [124].

### 6.2. Receptor-Mediated Targeted Treatment Strategies for HSC and Clinical Translation

Hepatic stellate cells (HSC) express surface markers, such as PDGF receptors, which facilitate the HSC-targeted delivery of therapeutic cargo to mitigate liver fibrosis and its associated complications, such as portal hypertension and HCC [125,126,127,128,129,130,131,132]. HSC-targeted approaches may enable the selective delivery of drugs, oligonucleotides, and therapeutic peptides by enhancing precision and minimizing systemic toxicity. The delivered drugs may include chemical compounds or antibodies with anti-fibrotic, anti-proliferative, anti-inflammatory, and pro-apoptotic properties, as well as collagen synthesis inhibitors, tyrosine kinase inhibitors, and angiotensin inhibitors. Oligonucleotide-based therapies include small interfering RNA (siRNA), self-amplifying RNA (saRNA), and CRISPR-based approaches for gene silencing or activation [133].

PDGFR plays a critical role in liver fibrosis and is highly expressed in activated HSC. PDGFR-recognizing peptides (PPB) can facilitate the targeted delivery of anti-fibrotic biologics, such as interferon (IFN)-mimicking peptides containing IFN signaling domains, thereby enhancing HSC-specific uptake and therapeutic efficacy while minimizing systemic side effects. This approach has shown significant inhibition of CCl4-induced liver fibrosis in murine models [134]. These strategies present novel therapeutic opportunities for the treatment of liver fibrosis, other fibrotic diseases, and liver cancer. Additionally, these peptides could also serve as diagnostic tools for monitoring the progression of fibrosis.

In addition to PDGFR, other receptors implicated in HSC targeting include integrins, the mannose-6-phosphate/insulin-like growth factor II receptor (M6P/IGF-IIR), fibroblast growth factor receptor 1 (FGFR1), the elastin receptor RXFP1, retinol (vitamin A)-binding protein (RBP) receptors, type VI collagen receptors, low-density lipoprotein receptors (LDLR), synaptophysin (SYN), cluster of differentiation 44 (CD44), and the tumor necrosis factor-related apoptosis-inducing ligand receptor (TRAILR) [135]. SYN is a membrane glycoprotein expressed on differentiated portal fibroblasts. SYN is advantageous over other markers for the targeted delivery of anti-fibrotic agents because it forms part of endocytosing vesicles, leading to an increased likelihood of endocytosis of its ligand and associated cargo. Studies have shown that a single-chain antibody (scAb) C1-3 binds to SYN on activated HSC but not on hepatocytes, supporting the feasibility of using C1-3 scAb as a targeting ligand for HSC-specific delivery [136]. One potential limitation of this strategy is the nonspecific delivery to neuroendocrine and neural cells, although there is no evidence showing that scAb can cross the blood–brain barrier. An off-target effect was observed in the M6P targeting approach. In one study, particles containing M6P-FITC-dextran were incubated with different types of liver cells. Surprisingly, the particles were absorbed not only by HSC but also by Kuppfer cells and hepatocytes [137].

Several specific receptor-recognizing peptides or siRNAs have been designed to target these receptors for anti-fibrosis treatments. The insulin-like growth factor 2 receptor (IGF2R) is a multifunctional receptor that is overexpressed on activated HSC and serves as a specific molecular marker of activated HSC in the fibrotic liver. An IGF2R-specific peptide significantly increases the binding affinity and uptake of a protein-based siRNA nanocomplex by activated HSC, and the nanocomplex significantly improves the serum stability and silencing activity of siRNA, which has the potential to be a useful delivery system for specifically delivering therapeutic siRNAs to activated HSC in the fibrotic liver [138,139,140].

Hsp47 is a collagen chaperone, and its inhibition not only alters collagen expression and alignment but also promotes HSC death due to intracellular collagen misfolding. HSC-selective delivery of Hsp47 siRNA via vitamin A-coupled liposomes has shown promising results in a rat model of cirrhosis [132]. Phase 2 clinical trials were initiated in patients with advanced fibrosis or compensated liver cirrhosis who had eradicated HCV infection or MASH and compensated liver cirrhosis. In patients with eradicated HCV infection and F3-F4 fibrosis (NCT03420768), BMS-986263 achieved an improvement in fibrosis by ≥1 stage in 17–21% of cases compared to 13% in the placebo group, along with a reduction of HSP47 mRNA in most patients in the higher dose group. However, the effects of BMS-986263 on target gene expression were disappointing, showing only a reduction of 5.9% in HSP47 mRNA and 10.1% in HSP47 protein levels [141]. Notably, the phase 2 trial of BMS-986263 in patients with compensated MASH cirrhosis was terminated due to a lack of efficacy (NCT04267393). It is possible that the low target gene reduction, possibly due to suboptimal delivery to HSC, contributed to this low efficacy.

Targeted delivery of anti-fibrotic agents to activated HSC is critical for the successful treatment of liver fibrosis. A number of clinical trials have been conducted for the treatment of liver fibrosis using small molecules, proteins, monoclonal antibodies, and nucleic acids. Among these, one clinical trial (NCT02227459) used vitamin A as a targeting ligand for anti-fibrotic siRNA [140].

Currently, most investigators focus on natural ligands, which are very limited and generally have low affinity for these receptors. In addition, some of the natural ligands may activate downstream signaling pathways after binding to their receptors on HSC. A potential solution to this challenge is to use affinity selection technologies, such as phage display biopanning to discover peptide- or antibody-based ligands, or SELEX to discover aptamer-based ligands. Compared with natural ligands, artificial ligands—including peptides, antibody fragments, antibodies, and aptamers—exhibit higher affinity and greater flexibility for chemical modification and coupling to anti-fibrotic agents or delivery systems. In particular, peptides and aptamers are attractive ligands because of their small size, ease of production, and lack of immunogenicity.

### 6.3. Design and Progress of Peptide Drugs

Certain peptides target activated hepatic stellate cells, which are compounds formed by linking multiple amino acids through peptide bonds, such as specific receptors on HSC, including PDGFR, TGF-βR, and integrin receptors. The advent of the 21st century ushered in a new era in peptide drug development, with advancements in structural biology, recombinant biotechnology, and new synthesis and analytical technologies playing a significant role in accelerating this process. This has led to the establishment of a comprehensive peptide drug development framework, encompassing peptide drug discovery, design, synthesis, structural modifications, and activity evaluation. Peptide drugs offer advantages such as high specificity, selectivity, and potent efficacy at low concentrations. With the continuous development of structural biology and proteomics, many protein–protein interactions (PPIs) have been studied, involving cellular pathways and biological functions [142,143], as well as the regulation of signaling pathways related to physiological and pathological processes [144]. Compared to small molecule drugs, peptides exhibit stronger target affinity, higher specificity, and fewer side effects. In contrast to large molecule drugs, peptides demonstrate lower immunogenicity and reduced production costs. Thus, the rational design of peptides based on known crystal structures is regarded as a promising strategy for discovering and developing new peptide drugs. To date, more than 80 peptide drugs have received FDA approval for clinical use [145]. Liraglutide, a synthetic analog of human glucagon-like peptide 1 (GLP-1), is a successful example of peptide drug development and is widely used in the treatment of obesity and type 2 diabetes [146,147]. This success in peptide drug development provides valuable insights for targeted HSC therapies in the treatment of fibrosis, cirrhosis, and liver cancer.

## 7. Conclusions

In summary, there is currently a lack of direct anti-fibrotic or anti-cirrhotic therapeutic agents that have demonstrated clinical success. Identifying reversible tipping points in liver fibrosis/cirrhosis, establishing ideal diagnostic biomarkers or methods for non-invasively assessing the staging, progression, and reversal of liver fibrosis/cirrhosis, developing effective anti-fibrotic/cirrhotic therapies, and devising methods to prevent or treat tumors by improving the tumor microenvironment represent key challenges and frontiers in current research. Recent advances in omics, particularly single-cell sequencing and spatial transcriptomics, hold promise for identifying new HSC targets for the diagnosis and treatment of liver fibrosis/cirrhosis and liver cancer. Establishing specific HSC-targeted delivery methods is a critical step in translating these discoveries into effective clinical diagnostic and therapeutic strategies.

## Figures and Tables

**Table 1:** Representative clinical trials of potential anti-fibrotic therapies in chronic liver diseases.

HSC Activation Target	Drug Name	Drug Category	Mechanism	Population (*n*)	Study Start Year	Highest Status (Phase)	NCT	Status
TGF-β/TGFβR	Hydronidone	Small molecule	Inhibitor	Liver fibrosis (248)	2021	III	NCT05115942	Completed
αvβ6 Integrin	PLN-74809	Small molecule	Inhibitor	Primary sclerosing cholangitis(121)	2020	II	NCT04480840	Completed
FGF21	BIO89-100	Fusion proteins	Activator	MASH (101)	2019	II	NCT04048135	Completed
	Efruxifermin	Fusion proteins	Activator	MASH (110)	2019	II	NCT03976401	Completed
	Pergbelfermin	Fusion proteins	Activator	Liver cirrhosis and MASH (155)	2018	II	NCT03486912	Completed
FGF19	Aldafermin	Fusion proteins	Activator	MASH (171)	2019	II	NCT03912532	Completed
PDGF/PDGFR	Imatinib	Small molecule	Inhibitor	Liver fibrosis (20)	2021	II	NCT05224128	Unknown status
WNT/β-catenin	PRI-724	Small molecule	Inhibitor	Liver cirrhosis (27)	2018	II	NCT03620474	Completed
LOXL2	PXS-5382A	Small molecule	Inhibitor	MASH (18)	2019	I	NCT04183517	Completed
PPARα/γ	Saroglitazar	Small molecule	Activator	MASH (20)	2019	II	NCT03639623	Completed
PPARα/δ/γ	Lanifibranor	Small molecule	Activator	MASH (1000)	2021	III	NCT04849728	Recruiting
PPARα	Pemafibrate	Small molecule	Activator	MASH (118)	2017	II	NCT03350165	Completed
PPARα/δ	ZSP0678	Small molecule	Activator	MASH (104)	2019	I	NCT04137055	Completed
TLR4	JKB-121	Small molecule	Inhibitor	MASH (65)	2015	II	NCT02442687	Completed
	JKB-122	Small molecule	Inhibitor	MASH (300)	2020	II	NCT04255069	Unknown
LOXL2, PDE3/4	Epeleuton	Small molecule	Inhibitor	MAFLD (96)	2016	II	NCT02941549	Completed
AMPK	PXL-770	Small molecule	Activator	MAFLD (211)	2019	II	NCT03763877	Completed
MMP(MMP2,MMP9, VEGF-A)	ALS-L1023	Small molecule	Inhibitor	MASH (60)	2019	II	NCT04342793	Completed
FXR	Obeticholic Acid	Small molecule	Inhibitor	MASH (2477)	2015	III	NCT02548351	Terminated
	Tropifexor	Small molecule	Inhibitor	MASH (234)	2019	II	NCT04065841	Terminated
HSP47	BMS-986263	siRNA	Inhibitor	MASH (124)	2021	II	NCT04267393	Terminated
GLP-1 receptor	Semaglutide	Small molecule	Activator	MASH (1200)	2021	III	NCT04822181	Active, not recruiting
GLP-1/GIP receptor	Trizepatide	Small molecule	Activator	MASH (190)	2019	II	NCT04166773	Completed
GLP-1/Glucagon receptor	Cotadutide	Small molecule	Activator	MASH (54)	2019	II	NCT05364931	Completed
GLP-1/GIP/Glucagon	HM-15211	Small molecule	Activator	MASH (240)	2020	II	NCT04505436	Recruiting
THRβ	Resmetirom	Small molecule	Activator	MASH (1759)	2019	III	NCT03900429	Active, not recruiting
	VK2809	Small molecule	Activator	MASH (248)	2019	II	NCT04173065	Completed
MPC	Azemiglitazone	Small molecule	Activator	MASH (1800)	2022	III	NCT03970031	Unknown
	Deuterium-stabilized (R)-Piglitazone	Small molecule	Activator	MASH (117)	2022	II	NCT04321343	Completed
PDEs (mainly PED2)	NZSP1601	Small molecule	Inhibitor	MASH (37)	2020	II	NCT04140123	Completed
A3AR	Namodenoson	Small molecule	Activator	MASH (60)	2017	II	NCT02927314	Completed
FASN	TVB-2640	Small molecule	Inhibitor	MASH and MAFLD (2000)	2025	III	NCT06692283	Not yet recruiting
Stem cell	HepaStem	Cell transplant therapies	Activator	MASH (23)	2019	II	NCT03963921	Completed

Note: data source: https://clinicaltrials.gov (accessed on 23 March 2025). Abbreviations: A3AR, Adenosine A3 Receptor; AMPK, AMP-Activated Protein Kinase; FGF, Fibroblast Growth Factor; FASN, Fatty Acid Synthase; GLP-1 Receptor, Glucagon-Like Peptide-1 Receptor; GIP Receptor, Gastric Inhibitory Polypeptide Receptor; HSP47, Heat Shock Protein 47; LOXL2, Lysyl Oxidase-Like 2; MASH, Metabolic Dysfunction-Associated Steatohepatitis; MAFLD, Metabolic Dysfunction-Associated Fatty Liver Disease; MMP, Matrix Metalloproteinases; MPC, Mitochondrial Pyruvate Carrier; PDEs (Mainly PDE2), Phosphodiesterases (Mainly Phosphodiesterase 2); PDGF, Platelet-derived Growth Factor; PDGFR, Platelet-derived Growth Factor Receptor; PPAR, Peroxisome Proliferator-Activated Receptor; TGF-β, Transforming Growth Factor Beta; TGFβR, Transforming Growth Factor Beta Receptor; THR, Thyroid Hormone Receptor; TLR4, Toll-Like Receptor 4; VEGF-A, Vascular Endothelial Growth Factor A; WNT/β-Catenin, Wingless/Integrated-Beta Catenin Signaling Pathway.

## Data Availability

Not applicable.
